# Policies to address Israel’s physician workforce challenges

**DOI:** 10.1186/s13584-026-00788-7

**Published:** 2026-10-28

**Authors:** Revital Sheinberg, Alon Kadmon, Alexey Belinsky, Ayelet Grinbaum Arizon, Rachel Berner Shalem, Bruce Rosen, Yifat Irshai Bas, Joseph Mendlovic

**Affiliations:** 1 Shamir Medical Center, Zerifin, Israel; 2 Ministry of Health, Jerusalem, Israel; 3 Hebrew University, Jerusalem, Israel; 4 Shaare Zedek Medical Center, Affiliated with the Hadassah-Hebrew University School of Medicine, Jerusalem, Israel; 5 The Israeli Ministry of Health - Office of the Associate Director General, Yermiyahu 39, Jerusalem, 913101 Israel

## Abstract

**Background:**

Israeli policymakers have four key concerns about Israel’s future physician workforce: an insufficient number of physicians, an uneven quality of training, a geographic maldistribution, and a sub-optimal specialty mix. Recently, Israeli policymakers have launched a multi-year, multifaceted effort to address those workforce challenges. This comprehensive review of the policy responses seeks to fill a gap in the English-language literature, making it possible for scholars worldwide to contribute to the development of effective Israeli workforce policies and their evaluation.

**Methods:**

This integrative article was written by a group of senior and mid-level professionals based at the Ministry of Health, all of whom are significantly involved in healthcare workforce issues. In writing the article, the authors drew on a wide range of official government reports, internal government memos, journal articles, OECD reports, Israeli and OECD databases, and Israeli committee reports.

**Findings:**

The policy responses fall into the following six clusters: 1. Implementing strategic workforce planning and data-informed workforce regulation. 2. Increasing the number of students trained annually in Israeli medical schools. 3. Increasing the supply of well-trained physicians from non-Israeli medical schools. 4. Streamlining and modernizing post-graduate medical education. 5. Increasing the number of young people from the geographic periphery who successfully pursue medical careers. 6. Expanding training and employment opportunities for advanced practice professionals. The multi pronged workforce modernization agenda that Israel is now implementing—at remarkable speed and with substantial budgetary resources—is similar to that which many OECD countries have been building gradually over the past decade. It has also been adapted to Israel’s unique demographic pressures, reliance on foreign trained physicians, and longstanding geographic disparities.

**Conclusions:**

Successfully addressing Israel’s many workforce challenges will require a stepping up of the analysis, creativity, resourcefulness, cooperation and budgetary expansion that have characterized recent efforts to meet Israel’s medical workforce needs. To maximize the impact of Israel’s multi-faceted workforce reform, it will also be important to solicit ongoing input from national and international workforce experts.

**Supplementary Information:**

The online version contains supplementary material available at https://doi.org/10.1186/s13584-026-00788-7.

## Introduction

Israel faces substantial and multifaceted challenges regarding its physician workforce, drawing concern from policymakers over the health system’s ability to meet the needs of the country’s population [1–10]. Over the past decade, these concerns have crystallized around four key interrelated challenges: an uneven quality of training, an inadequate overall quantity of physicians, geographic maldistribution, and a sub-optimal specialty mix.

First, there is an uneven quality of training among the medical workforce [10]. Israel has a limited domestic medical school capacity and has historically relied heavily on foreign medical schools [4]; by 2023, nearly 70% of newly licensed physicians were trained abroad [11]. Many of these individuals studied in unrecognized foreign institutions that offered inadequate clinical exposure [12, 13]. Graduates from these unrecognized schools have exhibited significantly lower pass rates on the Israeli medical licensure exam, and those who do pass often face knowledge gaps and challenges securing residency positions [14].

Second, Israel faces an impending inadequate quantity of physicians [5, 7, 8]. While the country historically maintained its physician supply through waves of immigration and overseas training, the system is now strained by one of the fastest-growing and aging populations in the OECD [4, 15, 16]. This rising demand is colliding with the impending retirement of an aging physician workforce. Furthermore, the recent “Yatziv reform”—which disqualified unrecognized foreign medical schools in order to address quality concerns—is expected to reduce the annual number of new medical license recipients by up to a third [2]. A recent trend toward reducing the weekly work hours of hospital residents has further increased the systemic need for more physicians [7]. 

Third, the health system suffers from a pronounced geographic maldistribution [17]. The physician workforce is disproportionately concentrated in the center of the country. For example, the Tel Aviv metropolis recently averaged 5.5 physicians per 1,000 residents, compared to just 2.7 in the northern and southern peripheries [18]. This gap is qualitative as well as quantitative; peripheral areas rely much more heavily on graduates from unrecognized foreign medical schools, as domestically trained physicians typically prefer the professional and personal benefits of working in the country’s center [19, 20]. 

Finally, Israel struggles with a sub-optimal specialty mix [7, 21]. The system experiences acute shortages of residents and board-certified specialists in fields such as psychiatry, rehabilitation, and pediatrics, alongside surpluses in other areas. These imbalances are driven by factors that include burdensome workloads, limited private practice opportunities, and a decentralized system where hospitals determine residency slots based on immediate staffing needs rather than national long-term planning [10, 21, 22].

Addressing these four systemic challenges has become a critical priority for the Israeli Ministry of Health (MOH). Recently, policymakers have launched a multi-year, multifaceted effort to stabilize and modernize the medical workforce. This article provides a comprehensive review of these policy initiatives, exploring how strategic planning, educational expansion, and targeted incentives are being deployed to ensure Israel has an adequate, well-trained, and equitably distributed physician workforce for the future. Its primary objective is to updates scholars and policy experts, in Israel and beyond, about recent Israeli policies and programs and encourage them to contribute to the development and evaluation of effective workforce policies in Israel.

## Methods

This integrative article was written by a group of senior and mid-level professionals based at the MOH, all of whom are significantly involved in healthcare workforce issues. In writing the article, they drew on a wide range of official government reports, internal government memos, journal articles, OECD reports, Israeli and OECD databases, and Israeli committee reports.

## Findings

Israeli policymakers have launched a major, multi-year and multifaceted program to ensure that in the years ahead Israel will have an adequate supply of well-trained physicians throughout the country,

Most of the changes underway fall into six clusters, as follows:

1. Implementing strategic workforce planning and data-informed workforce regulation.
2. Significantly increasing the number of students trained annually in Israeli medical schools.
3. Streamlining and modernizing post-graduate training.
4. Increasing the supply of well-trained physicians from non-Israeli medical schools.
5. Increasing the number of young people from the periphery who successfully pursue medical careers.
6. Expanding training and employment opportunities for advanced practice professionals.

Table 1 highlights which of the 6 policy clusters address each of the 4 workforce challenges. As indicated in the table, all of the challenges are addressed by at least two of the policy clusters, with the quantitative and qualitative challenges being addressed by the greatest number of policy clusters. Most of the policy clusters address two of the challenges, and one of the clusters (Implementing strategic workforce planning) addresses all four challenges.

The large number of policy clusters and the even larger number of specific policy initiatives reflect the diversity and difficulty of the challenges to be addressed. They also reflect uncertainty regarding the likely impact of many of the new (and often novel) policy initiatives. This underscores the need for future efforts to measure their individual and collective impacts.

**Table 1:** The primary links between the policy clusters and the challenges

	The uneven quality of training	The overall insufficient quantity	The geographic maldistribution	The sub-optimal specialty mix
Implementing strategic workforce planning and data-informed workforce regulation	X	X	X	X
Significantly increasing the number of students trained annually in Israeli medical schools	X	X		
Increasing the supply of well-trained physicians from non-Israeli medical schools	X	X	X	
Streamlining and modernizing post-graduate medical education	X	X		
Increasing the number of young people from the periphery who are successfully pursuing medical careers			X	
Expanding training and employment opportunities for advanced practice professionals	X	X		X

### Cluster #1: Implementing strategic workforce planning and data informed workfשגגןorce regulation

The need for Israel to engage in medical workforce planning has long been acknowledged. As detailed in Supplemental File 1, over 10 committees and reports have recommended it over the past 20 years. In July 2023, an OECD report specifically addressed this issue and advised Israel to transition from workforce crisis management towards establishing a more enduring structure for long-term planning. Additionally, it recommended moving from a largely free-market model toward a more structured national approach to postgraduate residency planning, including the development of national targets for the number and composition of specialists and a framework for aligning residency training with these targets.reEven before the OECD report, in 2022 the MOH had begun to establish a national infrastructure for health workforce planning. In 2023, in the wake of the OECD report, health workforce planning even became a central focus of the MOH’s top leadership and was given a prominent place in the MOH’s multi-year strategic plan.

Accordingly, the MOH has invested in improving its databases on physicians and other health professionals and in creating a Human Resources Planning Division dedicated to serving as the national authority for workforce planning in Israel. The department’s mandate covers planning for both physicians and other health professions.

Regarding physicians, there are two main branches of activity. The first branch focuses on specialty planning. It sets objectives and forecasts the future need for specialists in each medical field. To this end, the MOH is developing a planning model based on evolving data sources. As part of this work, a permanent advisory body was established, comprising representatives from hospitals, health management organizations (HMOs), the Israel Medical Association’s Scientific Council, and relevant professional associations. During the first half of 2026, this body recommended targets for the number of specialists in most medical fields, with priority given to those facing severe shortages.

The second branch deals with policies regarding the overall number of physicians. It addresses general shortages and geographic gaps between central and peripheral areas. Activities include initiatives to increase the number of medical students, encourage Jewish physicians from abroad to immigrate to Israel, encourage Israeli physicians studying abroad to return, and expand programs aimed at boosting the number of physicians and residents in underserved areas. These programs are detailed below.

It is important to note that establishing an effective framework for strategic physician specialty planning in Israel poses a considerable challenge, given the predominantly free-market nature of medical specialty choice and the resistance from the medical association and medical professionals to imposing limitations on that choice. The open-market dynamic has resulted in a situation where crucial knowledge about the demand for physicians and services is predominantly held by healthcare providers (primarily hospitals and health plans) rather than the MOH. Additionally, because residents are the primary workforce in hospitals and hiring is directed by departmental operational needs, hospitals are concerned that national workforce planning would compromise their autonomy.

Recognizing these challenges, the MOH has emphasized that representatives from a wide variety of health sector organizations will be deeply involved the planning process, especially during the initial years of implementation.

An important distinction is therefore made between national workforce planning and direct centralized control of residency positions. Questions regarding the appropriate balance between national direction and provider autonomy arise across many of the interventions described in this article, but they are particularly important in specialty training, where Israel has historically had no centralized system for allocating residency positions. The current approach is therefore to establish national specialty and geographic workforce targets while relying initially primarily on financial and organizational incentives to encourage hospitals and health plans to expand or redirect training capacity toward priority fields and underserved areas. This is especially relevant when creating additional positions, rather than reallocating existing ones. The effectiveness of this approach will be evaluated over time, and additional regulatory tools may be considered if incentives alone prove insufficient.

A tentative goal for training physicians in Israel, which was established using the limited MOH data and the perceived teaching capability of hospitals and medical schools in Israel, is thatby the year 2030 Israel will be able to grant 1,700 new licenses each year to physicians who had studied in Israeli medical schools [1]. This target is expected to evolve as Israel develops a more sophisticated planning model.

As part of the process of establishing physician workforce targets for 2035, the MOH conducted a multi-stage workforce planning exercise that combined quantitative analyses of future physician supply and demand with a qualitative assessment of key characteristics of the healthcare system. The process was based on the development of specialty-specific workforce projections using data on physician licensing, employment, residency training, demographics, and healthcare utilization. In addition, in-depth interviews were conducted with key stakeholders across the healthcare system, and discussions were held within an advisory committee comprising representatives from hospitals, health plans, the Israeli Medical Association, the Scientific Council, and other relevant organizations. The integration of these data sources and perspectives enabled the formulation of long-term specialist workforce targets, taking into account future population health needs, geographic disparities, and specialty-specific training and employment patterns.

Regarding the physician-to-population ratio, the MOH’s initial goal is to keep it from falling dramatically as a result of the Yatziv Reform. Subsequently, the goal will be to increase this ratio to the OECD average.

### Cluster #2: Significantly increasing the number of physicians trained annually in Israeli medical schools

- Enhancing the level of cooperation between the MOH and the CHE

The first step taken by the MOH was to collaborate with Israel’s Council for Higher Education (CHE) to develop an action plan for increasing the throughput of Israeli medical schools and reducing Israel’s reliance on graduates of foreign medical schools.

b. Increasing the number of Israelis studying in Israeli medical schools

The government is working to increase the number of Israelis studying in Israeli medical schools by increasing the capacity for clinical rotations sites, increasing the capacities of existing Israeli medical schools, and reducing the number of non-Israelis studying in those medical schools.

In 2023, the government set and exceeded a goal that by 2024 the number of students starting medical school would be increased to 1,200 students (compared to the 823 in 2020), in keeping with the recommendations of key planning committees [1, 2]. In 2024, 1,286 new students started their studies. A new target for 2027 has been set at 1,700 new students.

The biggest barrier to increasing the number of medical students has been the availability of sites for clinical rotations. Accordingly, a Joint CHE and MOH survey was undertaken to assess the availability of clinical training sites. It revealed significant potential to increase the number and size of training sites, and hence increasing the annual number of new Israeli medical students.

A regulation was introduced in April 2022 providing incentives to boost the number of clinical field weeks conducted in hospitals. This initiative was incorporated into the national budgeting model, and it was part of the accounting regulations governing transactions between hospitals and health funds [23]. The law ties a specific annual budget allocation to the quantity of clinical weeks completed within hospitals, thereby encouraging hospitals to expand the total number of clinical weeks they offer.

In order to also increase the available clinical fields in community clinics, in 2022 the MOH began efforts to provide incentives to the health funds to expand clinical training for medical students in the community, beyond the training of family medicine residents. Other objectives of this intervention include establishing robust academic departments in the community to foster research expansion.

In further cooperation with the Committee for Planning and Budgeting, a body within the CHE, the MOH is taking steps to subsidize construction and renovations within medical schools to facilitate an increase in the number of medical students. This subsidy is still in the planning and development stages [2]. 

Another effort involves prioritizing Israeli medical students over foreign students’ programs. The three foreign student programs in Israel offered by Tel Aviv University, Ben Gurion University, and the Technion, collectively trained approximately 130 American students each year. Thus, many American students were trained to become physicians, while hundreds of Israelis were pushed to study medicine abroad [24]. 

A directive issued by the MOH, published in December 2022, prioritized allocating clinical fields in Israeli hospitals to domestic medical students. Training students from abroad (with private profits to hospitals) would only be allowed once the department has met the quota of 36 weeks per year intended for domestic students [25].

c. Establishing new Israeli medical schools

Three new medical school programs were established over the past few years. The Reichman University program – whose opening sparked public controversy as it is part of a private, for-profit institution^1^ – opened in the second semester of the 2024–2025 academic year. The programs at the University of Haifa and the Weizmann Institute (a relatively small MD-PhD program) opened in the 2025–2026 academic year.

#### Medical education in peripheral regions (northern and southern Israel)

Expanding medical education in Israel’s peripheral regions is an important component of national workforce planning. It may improve access to medical education for residents of these areas and strengthen local medical centers through greater exposure to teaching, students, and academic activity. However, expansion is constrained by a major bottleneck: the limited availability of clinical training sites, particularly in smaller hospitals in the north and south. Moreover, training students in peripheral regions does not by itself ensure that they will remain there to practice. Geographic retention therefore requires complementary policies, including the Ilanot, Ofakim, and Kochavim programs described elsewhere in this article.

In northern Israel, the Azrieli Faculty of Medicine in Safed has played an important role in this effort. Since its establishment in late 2011, it has expanded substantially, and in late 2023 opened a six-year medical program for the first time. This reflects a gradual increase in training capacity, alongside continued constraints in clinical placements and pre-clinical infrastructure. The opening of the University of Haifa School of Medicine in late 2025 further improved access to medical education for residents of northern Israel.

In southern Israel, medical education has also expanded and further growth is planned. Here too, clinical training capacity remains a key constraint. This is expected to improve substantially with the planned opening of the new hospital in the Be’er Sheva area after 2030, which should create additional capacity for clinical education and future expansion.

### Cluster #3:Streamlining and modernizing post-graduate training

Two major reforms are being implemented regarding post-graduate training: enhancing and shortening the internship year, and incorporating community-based clinical rotations as an integral component of hospital-based residency training.

#### Enhancing and shortening the internship year^2^

A reform of the internship year in Israel was launched in September 2025. This reform has two primary objectives: to improve the overall quality of the internship experience, and to shorten the internship period, thereby enabling new physicians to begin residency training more quickly.

As part of the quality improvement efforts, changes are being made to the number and structure of clinical rotations, targeted training programs are being introduced (with particular emphasis on graduates of unrecognized foreign medical schools), and a new system of supervision and feedback is being established. Furthermore, the standards for successful completion of the internship are being tightened. The guiding principle for all these changes has been to enable a smoother progression from medical studies to internship.

The shortening of the internship will occur in two phases. In September 2025, the internship period was reduced by three months. For 2027,the Ministry of Health will offer a further optional reduction of one to three months (at the discretion of the intern). As a result, the internship year is expected to be shortened from 12 months to 6 months.

Importantly, the shortening of the internship was introduced as part of a broader redesign of the internship rather than as a simple reduction in training time. The reform is intended to preserve, and potentially improve, the quality of clinical training through more focused rotations, strengthened supervision and feedback, and clearer completion standards. As the reform has only recently been implemented, its impact on clinical preparedness and readiness for residency will require ongoing evaluation.

#### Integration of community-based clinical rotations as an integral component of hospital-based residency training

As part of this reform, multi-month community-based clinical rotations will gradually be introduced in the following six specialties: pediatrics, geriatrics, adult psychiatry, child and adolescent psychiatry, obstetrics and gynecology, and orthopedics. These rotations are expected to provide improved training and better prepare residents to deliver care in community settings, which constitute the majority of healthcare services in Israel.

This initiative is scheduled to begin as a voluntary step in 2026 and will be implemented gradually until full mandatory integration is achieved across all six specialties.

#### Challenges ahead related to specialty training

There are further needs that have not yet been fully addressed, and there are plans to deal with them in the next stages. These include planning for subspecialties, which is scheduled for the next phase of the planning process after progress is made with the core specialties. Another priority area will be improving regulatory tools for implementing and guiding the number of residents.^3^

### Cluster #4: Increasing the supply of well-trained physicians from non-Israeli medical schools

As noted above, in recent years, around 50% of new physicians in Israel each year have been trained outside the country’s borders. Many of them studied in medical schools deemed to be of inadequate quality and it is important to upgrade their skill levels. Others studied in good quality medical schools, and with them the challenge is to promote their return to Israel. Many of the Israelis who studied abroad had first applied to Israeli medical schools but had not been accepted by them.

Looking to the future, with the unrecognized medical schools no longer a viable route to medical licensure in Israel it will be important to make it easier for Israelis to study in good quality medical schools abroad. It will also be important to encourage immigration to Israel of physicians who were born outside of Israel and who trained in good quality medical schools. In this section, we provide additional details on the initiatives related to each of those challenges.

The Ministry also considered whether selected overseas medical schools that did not initially meet the recognition criteria could improve sufficiently through guidance or partnership. In practice, however, the main deficiencies were often structural, particularly the absence of adequate clinical training sites and meaningful hospital affiliations. Several schools sought guidance on how to meet the required standards, but these core limitations could not be overcome. The Ministry therefore focused on defining minimum recognition standards and on supporting affected graduates, rather than formally sanctioning programs that lacked adequate clinical training infrastructure.

#### Upgrading the skill levels of Israelis who studied in unrecognized foreign medical schools

In 2023, the MOH launched three heavily subsidized training programs to support graduates of unrecognized foreign medical schools who began their studies before the implementation of the Yatziv Reform and hence are still permitted to take the Israeli licensing exam. These tailored programs reflect the Ministry’s recognition that, despite studying in institutions that did not meet the required standards, these individuals have made significant efforts and are on the path to becoming physicians in Israel. The MOH wants to give them the best possible opportunity to succeed, realize their potential, and integrate effectively into the healthcare system during internship, residency, and beyond, despite the suboptimal level of education they received.

The three programs correspond to key stages in the medical training process: before licensing, before residency, and during residency.

1. Clinical Completion Course (Pre-Licensing):

This program is aimed at candidates who have not yet passed the licensure exam. It is a one-year program based at the Sheba Medical Center that provides intensive clinical training to address educational gaps.

2. Internship Preparation Course (Post-Licensing):

This program is designed for graduates who have passed the Israeli licensure exam but face challenges securing residency positions—often due to clinical skill gaps and negative perceptions regarding the quality of their education. This group represents the largest among those addressed by the MOH programs, comprising several thousand physicians who graduated from unrecognized foreign medical schools. These individuals require not only additional clinical training, but also personalized support and development of “soft” skills to help them avoid being automatically disqualified or overlooked solely based on the institution where they studied.

The MOH provides funding to several hospitals offering this course, which is available in both 200-hour and 450-hour formats. Four hospitals have either completed or are currently conducting their first course cycles, with cohorts ranging from 9 to 22 participants. Following the initial round, it became evident that the courses in their current format do not sufficiently improve participants’ acceptance into residency. As a result, the program is now being revised to better meet the needs of this population.

3. Resident Support Program (During Residency):

The two hospitals with the highest proportion of residents from unrecognized schools received MOH funding to run a professional skills enhancement course for 20 residents in each cycle.

#### Encouraging a return to Israel of Israelis studying in recognized medical schools abroad

Some students who study in Europe and receive a European license to practice medicine may choose to stay and work in Europe. In order to encourage the return and integration of Israeli physicians who completed their medical studies overseas, several programs were established as follows:

To better support medical students studying abroad, in 2022 the MOH initiated voluntarily registration of such students. This initiative enables the authorities to maintain contact with Israeli students studying medicine abroad and to offer them specific benefits. For example, following a preliminary survey conducted in 2020, where medical students abroad expressed concerns about not receiving benefits from the National Insurance and the Income Tax Authority, the National Insurance Institute has been working for several years on drafting regulations to ensure that medical students abroad receive rights equal to those studying in Israel. Once these regulations have been adopted, only students registered as Israeli medical students studying abroad will be eligible for the benefit. To date, there is no guarantee that all Israeli medical students studying abroad have been registered, and there is an active effort to increase the number of registrations.

In addition, in 2024 the MOH began to subsidize clinical rotations in Israeli hospitals for Israelis who study abroad, during the fourth to sixth year of medical education. The practice of such students doing some of their electives in Israeli hospitals, mainly during summer or holiday vacations, is not new and is even quite common. However, until recently it was fully financed by the student. The current subsidy operates via a financing model where hospitals receive payment for every 4-week clinical rotation involving a minimum of 8 Israeli students from medical schools abroad. This program aims to enhance the students’ familiarity with the Israeli health system, enable them to acquire high quality clinical training, and help them to develop connections with clinicians in Israeli hospitals.

#### Providing financial support for Israelis studying abroad in good quality medical schools

Studying abroad is significantly more expensive than studying in Israel. As a result, some students forgo medical studies due to financial constraints. To address that issue, the “Ofakim” (“Horizons”) program, was initiated and funded in 2022–2023. Subsequently, the program was endorsed in the recommendations of the OECD’s 2023 report on Israel’s medical workforce planning, and it has been implemented effectively since 2024. The program provides financial support to Israeli students enrolled in recognized medical schools abroad. It is based on similar programs that have proven to be highly successful in Norway, Sweden, and several other countries (Lånekassen, 2025; ANSA, 2025).

This model serves as a loan program specially designed for medical students abroad, providing talented individuals with a highly subsidized government loan^4^ to pursue their studies at prestigious international universities. Through this program, agreements are established with foreign universities, and the government takes responsibility for repaying the loans of students who choose to specialize in much-needed clinical fields within the Negev and the Galilee regions. The clinical fields for residency which accredit the student loan repayment by the government will change annually in accordance with the specific need in Israel as determined by the MOH. In 2024, the first cohort of 73 students started their studies in this program.

#### Encouraging the immigration to Israel of physicians born and trained outside of Israel

Encouraging the immigration of elite physicians and other health professionals to Israel has been a priority for the MOH since 2022. In collaboration with other government ministries, a dedicated committee was established to formulate solutions to streamline the immigration process and licensing for medical professionals. The committee recommended measures to reduce bureaucratic hurdles and facilitate the study of Hebrew and required pre-immigration courses through online platforms. These recommendations have been accepted and are currently in development stages.

In addition, a joint venture between the MOH, the Ministry of Immigration and Absorption, and the NGO “Nefesh B’Nefesh” began operating in 2024 to encourage Jewish doctors and other medical professionals to immigrate to Israel. An event with more than 600 candidates from the health professions took place in New-York, and - for the first time - an event was held in Paris with more than 500 participants. Similar events across Europe and South America are to take place in the near future.

### Cluster #5: Increasing the number of young people from the periphery who successfully pursue medical careers

In an effort to strengthen healthcare services in remote and under-served areas, several initiatives have been launched that focus on attracting medical professionals to practice in these regions by providing incentives and opportunities for career development. These include programs for recent high school graduates (The Preparatory School), medical students (“Ilanot”), and hospital residents (“Northern Stars”, “Southern Stars”, and the “Ofakim” program mentioned above). In this section we provide short summaries of each of these three programs.

#### A pre-university preparatory school

Medical school graduates who grew up in central regions exhibit a strong preference for employment in central locations. Therefore, prevalence of central-Israel-born students in Israeli medical schools has adversely affected the demand for positions in hospitals and clinics located in peripheral areas.^5^

To increase the representation of periphery-born students in Israeli medical schools, a new strategy hopes to elevate the academic standards of these students. This approach will partially offset one of the primary factors contributing to the dominance of central-born students in medical schools – their higher socioeconomic status, which correlates with superior academic achievements in high school.

The Negev Development Authority and the Galilee Development Authority have jointly established and now fully fund a new pre-university preparatory school. This initiative aims to enhance the chances of Negev and Galilee residents gaining admission to medical studies. Participants in the program commit to working for a period of seven years in either the Galilee or Negev regions upon completing their studies. The Negev pre-academic preparatory school started its first course in 2023 and the Galilee school started in 2024.^6^

#### Ilanot – a program for medical students

To reduce the shortage of healthcare services in peripheral regions, the “Ilanot” program was introduced in 2022. This program grants 30 additional spots beyond existing quotas at Ben Gurion University and Bar Ilan medical schools, specifically for outstanding students from the Negev and the Galilee regions. As part of the selection process, the candidate’s willingness to work and stay in the peripheral area, and their willingness to help improve the medical services provided in those areas are taken into account.

The participants come from all areas in the country, but the intention of the MOH is to increase the likelihood that the students will remain in these regions after completing their studies. Participants in the program receive comprehensive non-academic training to strengthen their personal connection with the Negev and the Galilee. They also receive leadership training to encourage and prepare them for leadership positions in the periphery.

Full funding for their studies, including living expenses and academic assistance, is provided, and internship placements in these regions are also facilitated [27] .In return, the participants commit to work as physicians in the peripheral area for 7 years after graduation. The design of this program is based on international experience, which the OECD comprehensively reviewed in a working paper in 2014 [28]. 

#### Northern Stars and Southern Stars – a program for hospital residents

A collaboration of the faculties for medicine at Bar-Ilan University in Safed and Ben-Gurion University in the Negev and the Israeli lottery fund initiated the “Northern Stars” and “Southern Stars” program. This program identifies exceptional residents specializing in specific clinical fields who commit to practice in priority regions within Northern and Southern Israel. In return for their dedicated service, these residents receive research grants, dedicated time for research, mentoring, fellowship funding in Israel or abroad, and various other benefits.

The main idea behind the program is to avoid the adverse selection and waste that characterized earlier types of monetary grants [29] and to attract talented physicians to challenging residency fields and positions. The idea beyond the financial incentive is to create a prestigious alternative for the most-needed residencies and to develop future leaders and managers in those fields.

In 2023, the program was launched with 19 new residents specializing in psychiatry and anesthesiology. In 2024, it included 18 new residents, half specializing in psychiatry and the rest in obstetrics and gynecology, pediatrics, surgery, emergency medicine, forensic medicine, radiology, and internal medicine. In 2025, the program included 23 new residents in the fields of psychiatry, and in 2026 it is expected to include 40 new residents.

### Cluster #6: Increasing the use of advanced practice professionals

Despite all efforts to expand the availability of physicians in Israel, the MOH understands that it will be difficult to fully address the physician shortage solely by increasing the number of physicians. This is due to the country’s rapid population growth, the lengthy training period required to become a physician, and additional unpredictable factors such as wars and changing work patterns.

In addition, It is important to emphasize that the objective of these professions is both to address the shortage of physicians and to optimize the utilization of existing physicians. By delegating tasks that do not necessarily require a medical doctor to professionals from these newly developed fields, physicians can be redirected toward the clinical and professional responsibilities for which they were specifically trained.

Therefore, the Ministry of Health has chosen to act not only to increase the number and availability of physicians, but also to formally recognize new professions and train additional healthcare professionals who can support physicians and help expand their clinical capacity. Two such professions are presented here, physician associates (referred to as physician assistants in the US) and nurse practitioners. They have been in use for many years in other countries, but remain underdeveloped in Israel.

The contribution of advanced practice professionals may therefore extend beyond addressing the overall shortage of physicians. By expanding team-based clinical capacity, these professionals may help address service gaps in selected specialties and may also support access to care in underserved settings. However, particularly at this early stage of their development in Israel, they are not intended to substitute for an adequate physician workforce in peripheral regions. Given the more pronounced physician shortages in these regions, particular caution is warranted to ensure that these professions are introduced as a complement to physician care rather than as a lower-staffing alternative specific to underserved areas. Their expansion should therefore complement, rather than replace, efforts to recruit and retain physicians in these areas, while maintaining appropriate supervision and standards of care.

#### Physician associates (PA’s)

In Israel, physician associates will undergo specialized training to support physicians in specific tasks, optimizing physicians’ time and reducing attrition. They will work under the supervision of specialists, carrying out predefined tasks, following a hierarchical model similar to that in the US. In 2023, a significant step was taken when the Knesset passed a law extending some of a physician’s powers to physician associates. In 2024 the CHE approved the PA Master’s degree. Additionally, the scope of professions has been expanded to include PAs in the community who are authorized to write first prescriptions for specific medications.

These developments build upon the success of a pilot program in which paramedics underwent a one and a half-year training course to become physician associates in emergency departments. The pilot was deemed successful and laid the groundwork for advancing the profession. Today, approximately 100 physician associates from this program are working in Israeli emergency departments.

The first academic training track for physician associates is expected to be launched in 2026, aiming to deploy them into the workforce by 2029. Until then, the MOH is working to attract physician associates from abroad, mainly the US, to support the system during this transitional phase. These PAs are also expected to play a key role in establishing the PA profession within the Israeli healthcare landscape, and also contribute to their training.

#### Advanced practice nurses (i.e. nurse practitioners – NP’s)

In 2009, Israel recognized advanced practice nursing as an official professional role. Palliative care was the first specialty recognized for this role and emergency medicine is the most recent. Today, nurse practitioner specialties exist in palliative care, geriatrics, neonatology, diabetes, surgery, health policy and management, pain management, primary care, rehabilitation, wound care, psychiatry, pediatric intensive care, emergency medicine, oncology and hematology, and neurology. To date, 1,027 nurses have been trained in these fields.

The integration of nurse practitioners is relevant to both hospital and community settings. Their role enhances the capacity of the health care team and helps alleviate the burden on the healthcare system. They are authorized to manage simple acute conditions, provide continuous care for patients with chronic illnesses, including symptom management, and improve treatment adherence [30, 31]. 

The Ministry of Health’s Nursing Division aims to increase the proportion of nurses who are nurse practitioners from the current 1% to 6% by 2030. The main barrier to integrating nurse practitioners into the healthcare system is the limited number of designated positions. However, in departments where nurse practitioners have been integrated, their contribution has proven highly significant—both in improving quality of care and reducing pressure on physicians. As a result, the MOH has prioritized expanding nurse practitioner roles in areas with high demand for skilled medical personnel. The MOH has supported targeted programs in neonatal care, leading to the successful integration of a nurse practitioner in nearly every neonatal unit today, and also supports integration programs in psychiatric and emergency departments.

## Discussion

In recent years, Israel has launched a large number of policy initiatives to address its medical workforce challenges. The breadth and intensity of the change has been driven by enhanced understanding of the severity of the crisis, an extensive consultation with the OECD, and several commissions and working groups examining the nation’s workforce challenges.

The consultation with the OECD constituted an important step forward in Israeli policymakers’ appreciation of the value of cross-national learning. To be sure, even before the OECD consultation, Israeli workforce planners and leaders drew periodically on the experience of other countries. For example, in 2014 Israel’s Council of Higher Education appointed an ad hoc external committee, composed primarily of experts from the U.S. and the UK, to perform a review of Israel’s four medical schools[6, 32, 33]. In addition, in 2020 Israel’s Ministry of Health asked the Myers-JDC-Brookdale Institute to present up-to-date data on the regulations and work hours regarding medical residents in OECD countries [34]. Still, prior to the OECD consultation many key players in Israeli healthcare tended to see Israel’s workforce challenges as being exceptional, with limited opportunities for cross-national learning.

In the wake of the OECD consultation, that attitude has changed markedly. Israeli policymakers now appreciate that, over the past decade, many OECD countries have pursued workforce reforms which are similar to some of the reforms now unfolding in Israel [35, 36]. Across Europe, North America, and parts of the Asia-Pacific region, governments have expanded domestic medical-school capacity, strengthened accreditation and quality assurance for foreign-trained physicians, shifted more clinical training into community settings, and introduced structured, data-driven workforce planning to replace fragmented, market-driven approaches. Many OECD systems have also invested heavily in advanced practice roles—particularly nurse practitioners and physician associates—to relieve pressure on physicians and improve access in underserved areas.

Israel’s current reform program mirrors this trajectory in many ways: a rapid scale-up of medical-school enrolment, closure of low-quality foreign-student tracks, the Yatziv reform to ensure training quality abroad, major restructuring of internship and residency training, targeted incentives to improve geographic distribution, and accelerated development of PA and NP roles.

In effect, Israel is now implementing—at remarkable speed— much the same multi-pronged workforce modernization agenda that many OECD countries have been building gradually over the past decade, but adapted to Israel’s unique demographic pressures, reliance on foreign-trained physicians, and longstanding geographic disparities.

The Netherlands is one of the countries whose medical workforce reforms have had a substantial impact on Israel’s. The Israeli Ministry of Health examined the Dutch model during the process of formulating its medical workforce planning program and establishing the planning body. It adopted from this model the structure of the advisory council, particularly its independent status and its composition of diverse stakeholders within the system, in order to generate consensus around recommendations and to learn from field experience rather than carry out unilateral, top-down planning. (For further information on the Dutch reform see Supplemental file 2).

All this points to further opportunities for bi-directional cross-national learning. Among the lessons to be learned from Israel is the importance of looking at a broad range of workforce challenges in an integrated manner (as has been done in this article), as opposed to focusing on the challenges one at a time. In addition, other countries may be able to learn from innovative policy responses being implemented currently in Israel, such as the “Ofakim” program. Additional features of the Israeli reform that are of potential interest to other countries are the attempt to address quality and quantity issues simultaneously and the introduction of a large number of initiatives over a relatively short time period. Issues on which Israel can continue to learn from other countries include how best to monitor progress on workforce goals and how best to monitor the impact of key workforce interventions.

## Conclusions

Israel’s physician workforce faces challenges in terms of quantity, quality, geographic distribution, and specialty distribution. To address these challenges, Israeli policymakers have explored their causes and begun to implement an ambitious multi-pronged response. Successfully addressing the many challenges will require a stepping up of the analysis, creativity, resourcefulness, cooperation and budgetary expansion that have characterized recent efforts to meet Israel’s physician workforce needs.

Our article provides the first comprehensive English-language review of Israel’s major, multi-faceted policy and programmatic response to address those concerns. Several of the authors of this article are among the leaders of Israel’s efforts to address the challenges facing its physician workforce. They look forward to hearing from workforce experts - from Israel and from other countries - who are willing to contribute to that effort through suggestions, insights, and analyses. In the years ahead, international workshops and cross-national studies could illuminate how the Israeli experience can inform, and be informed by, parallel developments in other countries.

**Graph. 1:**
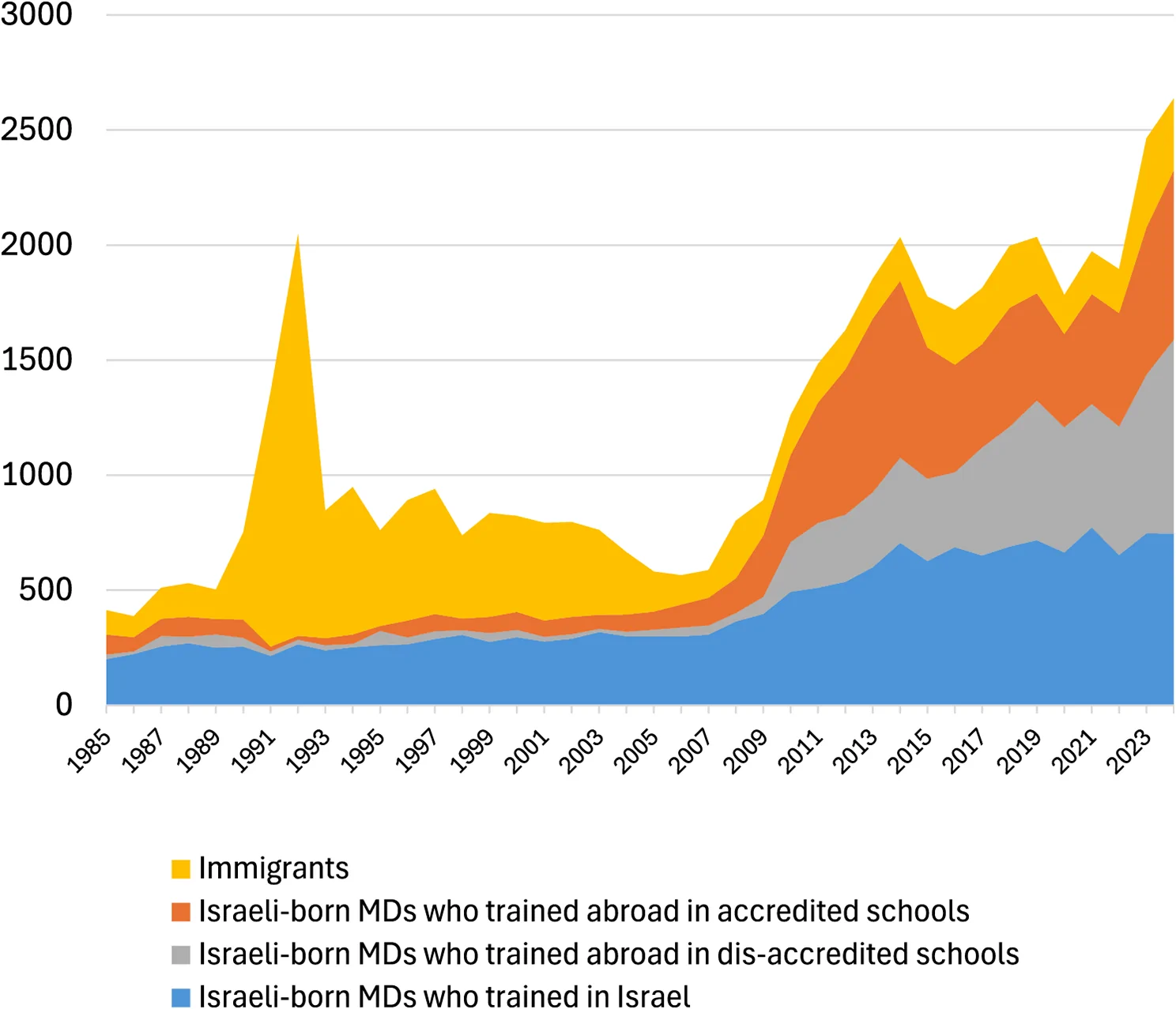
Newly licensed physicians by training background (four groups)

## Supplementary Information

Below is the link to the electronic supplementary material.

Supplementary Material 1

Supplementary Material 2

## Data Availability

The authors will consider requests to access any data and materials.

## Footnotes

1 Aside from Reichman, all Israeli universities are private non-profit institutions which are financed largely by government and in which tuition covers only a small part of total costs. In comparison with the other Israeli universities, tuition is substantially higher, and it covers a greater proportion of total costs.

2 Israel is among the countries in which the internship year is separate from residency training. In other countries, such as the US, the internship is a part of residency training as the PGY1 year

3 An incentive model is being developed, in line with the planning objectives, that will serve as the primary mechanism for implementation. Once it is completed and its effectiveness is evaluated, additional measures will be considered.

4 The loans are provided through a bank chosen via a tender process.

5 Legal barriers have hindered initiatives which aimed to restrict enrolment to students who lives or grew up in peripheral areas in programs such as “Ilanot”.

6 Although many countries around the world reserve university spots or give priority to residents of peripheral regions, this has not been possible in Israel due to the Student Rights Law, which prohibits any form of discrimination in university admissions to prevent political influence over acceptance decisions [26]. The Ministry of Health attempted to reserve spots in the “Ilanot” program exclusively for local residents—effectively creating a legal exception for a unique program aimed at prioritizing young people from the periphery. However, following legal review, this was ultimately not approved.
