# Supplementary material for: Policies to address Israel’s physician workforce challenges
Source: Isr J Health Policy Res. 2026 Oct 28;15:45. doi: 10.1186/s13584-026-00788-7 (PMC13715295; doi:10.1186/s13584-026-00788-7)
Supplement: Supplementary file 1 [file 13584_2026_788_MOESM1_ESM.docx]

**Supplemental file 2:
Medical workforce planning in the Netherlands**

Medical workforce planning in the Netherlands is facilitated through a sophisticated, demand-based simulation model managed by the Advisory Committee on Medical Manpower Planning (ACMMP, established in 1999). It is an independent body, that integrates representatives from medical professions, health insurers, and training institutions. The model was developed by NIVEL (the Netherlands Institute for Health Services Research), and it is designed to identify existing workforce gaps while projecting supply and demand requirements over 10- and 20-year horizons using multi-scenario analysis. on the supply side, it integrates empirical data regarding actual retirement patterns, international migration, and training completion rates. On the demand side, it accounts for demographic shifts, specifically population aging, as well as epidemiological trends and socio-cultural developments. The primary output of this process is a formal recommendation to the government on the annual student intake required to sustain labor market equilibrium. This planning mechanism has effectively achieved stakeholder consensus and stabilized the medical labor market, its predictive accuracy is maintained through continuous evaluation.

**Sources**

Sutton, C., Prowse, J., McVey, L., Elshehaly, M., Neagu, D., Montague, J., Alvarado, N., Tissiman, C., O’Connell, K., Eyers, E., Faisal, M., & Randell, R. (2023). Strategic workforce planning in health and social care – an international perspective: A scoping review. Health Policy, 132, 104827.

Van Greuningen, M., Batenburg, R.S. & Van der Velden, L.F. Ten years of health workforce planning in the Netherlands: a tentative evaluation of GP planning as an example. Hum Resour Health 10, 21 (2012). [https://doi.org/10.1186/1478-4491-10-21](https://eur02.safelinks.protection.outlook.com/?url=https%3A%2F%2Fdoi.org%2F10.1186%2F1478-4491-10-21&data=05%7C02%7CBruce%40jdc.org%7C13f2f746da4e46ea140708ded66ddfda%7C89e4c90de39c49dba2aaeeb7e02c7786%7C0%7C0%7C639183964597544156%7CUnknown%7CTWFpbGZsb3d8eyJFbXB0eU1hcGkiOnRydWUsIlYiOiIwLjAuMDAwMCIsIlAiOiJXaW4zMiIsIkFOIjoiTWFpbCIsIldUIjoyfQ%3D%3D%7C0%7C%7C%7C&sdata=UdPej9MIRvHSPiJzX%2FChzzLK38A7GZzTZW6pPfXHpPw%3D&reserved=0)

Ono, T., G. Lafortune and M. Schoenstein (2013), “Health Workforce Planning in OECD Countries: A Review of 26 Projection Models from 18 Countries”, OECD Health Working Papers, No. 62, OECD Publishing, Paris, [https://doi.org/10.1787/5k44t787zcwb-en](https://eur02.safelinks.protection.outlook.com/?url=https%3A%2F%2Fdoi.org%2F10.1787%2F5k44t787zcwb-en&data=05%7C02%7CBruce%40jdc.org%7C13f2f746da4e46ea140708ded66ddfda%7C89e4c90de39c49dba2aaeeb7e02c7786%7C0%7C0%7C639183964597569816%7CUnknown%7CTWFpbGZsb3d8eyJFbXB0eU1hcGkiOnRydWUsIlYiOiIwLjAuMDAwMCIsIlAiOiJXaW4zMiIsIkFOIjoiTWFpbCIsIldUIjoyfQ%3D%3D%7C0%7C%7C%7C&sdata=PtXmmMZv8mwya0qolLQXpzrdx8qXEYaKFV4zeAyNazw%3D&reserved=0).
