# Supplementary material for: Policies to address Israel’s physician workforce challenges
Source: Isr J Health Policy Res. 2026 Oct 28;15:45. doi: 10.1186/s13584-026-00788-7 (PMC13715295; doi:10.1186/s13584-026-00788-7)
Supplement: Supplementary file 2 [file 13584_2026_788_MOESM2_ESM.docx]

**Supplemental file 1:
Key recommendations of national committees regarding the physician workforce
and the extent to which the recommendations have been adopted**

| **Committee** | **Date** | **Key recommendations** | **Status** |
| --- | --- | --- | --- |
| Levi committee | 2007 | - Increase the number of medical graduates from Israeli medical schools to 600 per year - Establish an additional medical school in Israel - Create a 4-year medical degree track. | The committee's recommendations were adopted by the CHE and all the key recommendations have been implemented. |
| Bin-Nun committee | 2007 | - Expand medical school capacity in Israel to approximately 600 graduates per year - Review programs for foreign students and reallocate vacant spots to Israeli students. - Ensure appropriate budget allocations for medical schools. | As of today, the recommended number of Israeli medical school graduates has been achieved; the foreign students programs were cancelled prioritizing Israeli students.  Budgets have been increased to reflect the increase in the number of medical students, but there has not been a broader, more fundamental change to the basic budgets. |
| Horeb committee | 2010 | - Adopt a ratio of 2.9 physicians per 1000 population in 2025 as a minimum threshold for the Israeli healthcare system. - Increase the number of students beginning medical studies in Israel each year to 900 by 2014. - Encouraging the return to Israel of Israeli physicians living abroad, as well as Israeli medical students studying abroad. | As of today, the physician-to-population ratio exceeds 2.9 per 1,000, and the target of 900 students starting medical school annually has been met since 2021. Programs such as ‘Ofakim’ and the programs in the Physicians’ Department at the Ministry of Aliyah and Integration were launched to strengthen ties with foreign and Israeli doctors abroad. |
| Schoenbaum commission | 2014 | - Medical schools should enhance governance and the authority of deans. - The CHE should review English-language programs and the internship year. - Shift teaching from heavy reliance on lectures to interactive, small-group, and clinical learning with earlier and broader exposure, including community settings. - Focus assessments on clear competency goals, emphasize performance-based methods, and incorporate simulations instead of relying mainly on multiple-choice tests. - Strengthen faculty development, MD-PhD program structure, and infrastructure such as simulation centers - Improve national evaluation and workforce planning to meet Israel’s healthcare needs. | The foreign student programs have been closed, and a planning department at the Ministry of Health is working on workforce planning. As the MD degree is now awarded at the end of medical school — a decision made by the CHE — the internship year has become the responsibility of the MOH.  While specific medical schools have made some changes in teaching methods, there has not been a major system-wide effort to reform teaching methods. |
| Marom Halperin committee | 2018 | - Admit 70 students to the four-year program at Ariel’s Faculty of Medicine - Reduce or cancel programs for international students in favor of 55–130 additional Israeli students - Cancel the Nicosia program while adding 30 students at Tel Aviv University. - Expand the military track (Atuda) and Bar-Ilan University’s program by 20 students each. - Establish uniform affiliation agreements between faculties and hospitals - Increase budget allocations per student - Standardize compensation for teaching - Align of residency positions in core specialties with numbers of graduates - Update models for clinical education | The foreign student programs have been closed. In 2024, nearly 90 students began the four-year program at Ariel University, and in 2023 Bar-Ilan University expanded its six-year track by 80 students. As part of the incentive law for increasing the number of clinical fields in hospitals, a maximum price was set for agreements between hospitals and universities for clinical training sites, and a budget was allocated to establish teaching units in hospitals to improve teaching methods and outcomes.  There has not been a change in the number of students in the military medical program ‘Tzameret. The Nicosia program is still operating. Budgets have been increased to reflect the increase in the number of medical students, but there has not been a broader, more fundamental change to the basic budgets. |
| Gamzo committee | 2022 | - Increase the annual number of new medical licenses from 1721 in 2020 to 2,000 by 2035 - Increase the number of students in Israeli schools from 821 in 2020 to 1200 in 2025, through expanding the number of medical students in Israel. - Shorten the internship period. - Expand clinical training in the community and in specialties in shortage, and expand the capacity of clinical fields in hospitals. - Establish dedicated functions to manage medical education for students and residents, alongside incentivizing hospitals, departments, and physicians involved in teaching. - Encourage the return of Israeli students studying medicine abroad and senior Israeli physicians who have emigrated through tailored incentives, as well as subsidizing studies abroad for outstanding students in exchange for a commitment to work in the Negev, the Galilee, or in specialties in shortage. - Incentivize immigration of young specialist physicians. - Abolish programs for foreign students studying in Israeli medical schools. | In 2024, 2,637 new medical licenses were issued and 1,286 students began studies in Israeli medical schools, both exceeding committee targets. An internship reform is set to begin in September 2025, shortening the internship year by 3-5 months, and funding has been allocated for hospital teaching units to improve teaching in hospitals. The ‘Ofakim’ program was launched to support Israelis studying abroad, while the Ministry of Immigration and Absorption and Nefesh B’Nefesh helped bring 600 Jewish physicians last year, aiming to sustain this annually. Foreign student programs have been closed. For several years, the MOH has offered a support program that rewards universities for conducting clinical rotations in community clinics. It is well utilized in pediatrics and efforts are underway to expand its use in gynecology. |
| Biar and Halevy committee | 2022 | - Award the MD degree upon completion of medical studies. - Reform final exams into a single integrative theoretical and practical test. - Increase the number of medical students by 400 gradually over 4 years. - Increase clinical experience in community pediatrics and women’s health. - Integrate innovative teaching methods and technologies, including case-based learning, a personal practical internship during clinical training, simulations, competency-based syllabus, promoting student autonomy, and requiring a final thesis for graduation. - Grant the title of Clinical Instructor to residents involved in teaching. - Organize clinical rotations of 36–40 weeks per year with groups of 8–10 students - Prioritize Israeli students for clinical placements. | The MD degree is now awarded at the end of medical school. Between 2022 and 2024, Israeli medical student enrollment increased by 342 students annually and it appears to have exceeded 400 in 2025. Funding has been allocated to establish hospital teaching units to enhance teaching methods and outcomes. For several years, the MOH has offered a support program that rewards universities for conducting clinical rotations in community clinics. As part of the efforts to increase the number of clinical training sites in hospitals, the number and size of clinical rotations were reorganized and regulated.  While specific medical schools have made some changes in teaching methods, there has not been a major system-wide effort to reform teaching methods. The efforts to merge the exams into a single exam and to add a clinical component have not yet been completed. At present, there is no feasibility for direct compensation of resident instructors in hospitals. |
| OECD report | 2023 | - Shift from crisis management to a structured system of health workforce planning, similar to the Dutch model. - Urgently increase the number of Israeli medical students by expanding existing Israeli medical schools, opening new ones (including branches in the periphery), and closing programs for foreign students. - Broaden clinical training through additional shifts, larger groups, and rotations in community clinics. - Provide support to Israelis studying in accredited schools abroad. - Institute central planning of residency positions - Put in place a funded plan to financially support health institutions based on the number and type of training places offered in them for residents, according to the national planning. | The Ministry of Health has established a department for long-term workforce planning, which is working on multiple programs to increase the number of medical students; these programs have already produced results in recent years. The foreign student programs have been closed. Hospitals’ teaching units have been funded and are expected to open in the coming years, helping to improve the quality of clinical rotations. The ‘Ofakim’ program, which supports Israeli students studying in universities abroad, started in 2025. In addition, a model to incentivize hospitals for meeting training targets has been funded and is under development at the MOH.  For several years, the MOH has offered a support program that rewards universities for conducting clinical rotations in community clinics. |
